# Early‐Branching Cyanobacteria Grow Faster and Upregulate Superoxide Dismutase Activity Under a Simulated Early Earth Anoxic Atmosphere

**DOI:** 10.1111/gbi.70005

**Published:** 2024-12-12

**Authors:** Sadia S. Tamanna, Joanne S. Boden, Kimberly M. Kaiser, Nicola Wannicke, Jonas Höring, Patricia Sánchez‐Baracaldo, Marcel Deponte, Nicole Frankenberg‐Dinkel, Michelle M. Gehringer

**Affiliations:** ^1^ Department of Microbiology University of Kaiserslautern‐Landau RPTU Kaiserslautern Germany; ^2^ Department of Molecular Botany University of Kaiserslautern‐Landau RPTU Kaiserslautern Germany; ^3^ School of Geographical Sciences, Faculty of Science University of Bristol Bristol UK; ^4^ School of Earth and Environmental Sciences University of St. Andrews St. Andrews UK; ^5^ Plasma Bioengineering Leibniz Institute of Plasma Science and Technology Greifswald Germany; ^6^ Department of Chemistry University of Kaiserslautern‐Landau RPTU Kaiserslautern Germany

**Keywords:** Archean, climate change, cyanobacteria, oxygen, superoxide dismutases

## Abstract

The evolution of oxygenic photosynthesis during the Archean (4–2.5 Ga) required the presence of complementary reducing pathways to maintain the cellular redox balance. While the timing of the evolution of superoxide dismutases (SODs), enzymes that convert superoxide to hydrogen peroxide and O_2_, within bacteria and archaea is not resolved, the first SODs appearing in cyanobacteria contained copper and zinc in the reaction center (CuZnSOD). Here, we analyse growth characteristics, SOD gene expression (qRT‐PCR) and cellular enzyme activity in the deep branching strain, *Pseudanabaena* sp. PCC7367, previously demonstrated to release significantly more O_2_ under anoxic conditions. The observed significantly higher growth rates (*p* < 0.001) and protein and glycogen contents (*p* < 0.05) in anoxically cultured *Pseudanabaena* PCC7367 compared to control cultures grown under present‐day oxygen‐rich conditions prompted the following question: Is the growth of *Pseudanabaena* sp. PCC7367 correlated to atmospheric *p*O_2_ and cellular SOD activity? Expression of *sodB* (encoding FeSOD) and *sodC* (encoding CuZnSOD) strongly correlated with medium O_2_ levels (*p* < 0.001). Expression of *sodA* (encoding MnSOD) correlated significantly to SOD activity during the day (*p* = 0.019) when medium O_2_ concentrations were the highest. The cellular SOD enzyme activity of anoxically grown cultures was significantly higher (*p* < 0.001) 2 h before the onset of the dark phase compared to O_2_‐rich growth conditions. The expression of SOD encoding genes was significantly reduced (*p* < 0.05) under anoxic conditions in stirred cultures, as were medium O_2_ levels (*p* ≤ 0.001), compared to oxic‐grown cultures, whereas total cellular SOD activity remained comparable. Our data suggest that increasing *p*O_2_ negatively impacts the viability of early cyanobacteria, possibly by increasing photorespiration. Additionally, the increased expression of superoxide‐inactivating genes during the dark phase suggests the increased replacement rates of SODs under modern‐day conditions compared to those on early Earth.

## 1 Introduction

Life on Earth evolved under anoxic, slightly reducing conditions (Fischer and Valentine 2019; Hamilton 2019) and was predominated by anaerobic prokaryotes (Hamilton 2019; Ślesak et al. 2019). Molecular O_2_, a commonly used electron acceptor in aerobic respiration today (Stebegg et al. 2023), was not freely available (Kump 2008; Lyons, Reinhard, and Planavsky 2014). This changed upon the evolution of ancestral photosystems, capable of hydrolysing water and releasing O_2_, possibly during the late Archean (Nishihara et al. 2024; Cardona et al. 2019). Crown group cyanobacteria likely emerged ~3.2–2.8 Ga (Bianchini, Hagemann, and Sánchez‐Baracaldo 2024; Boden et al. 2021; Fournier et al. 2021), although other estimates exist (Shih et al. 2017). If microfossil calibrated estimates are true, a few hundred million years passed before the Earth's atmosphere was enriched with free O_2_ to 1% of present‐day levels (Sessions et al. 2009) during a period known as the Great Oxygenation Event (GOE), which began about 2.45 Ga (Bekker et al. 2004; Lyons, Reinhard, and Planavsky 2014; Warke et al. 2020). The gradual oxygenation of the environment resulted in the emergence of new enzymes and reaction pathways (Jabłońska and Tawfik 2021) and is thought to have caused large‐scale extinction of anaerobic lifeforms unable to cope with uncontrolled oxidation of their cellular components (Case 2017; Fischer, Hemp, and Valentine 2016; Fischer and Valentine 2019; Hamilton 2019). Free O_2_ would rapidly have been scavenged by reductants such as Fe(II), Mn(II) and ammonia or atmospheric volcanic gases (Ward, Kirschvink, and Fischer 2016); however, O_2_ levels in microbial mats may have exceeded present‐day atmospheric levels (Herrmann and Gehringer 2019).

**FIGURE 1:**
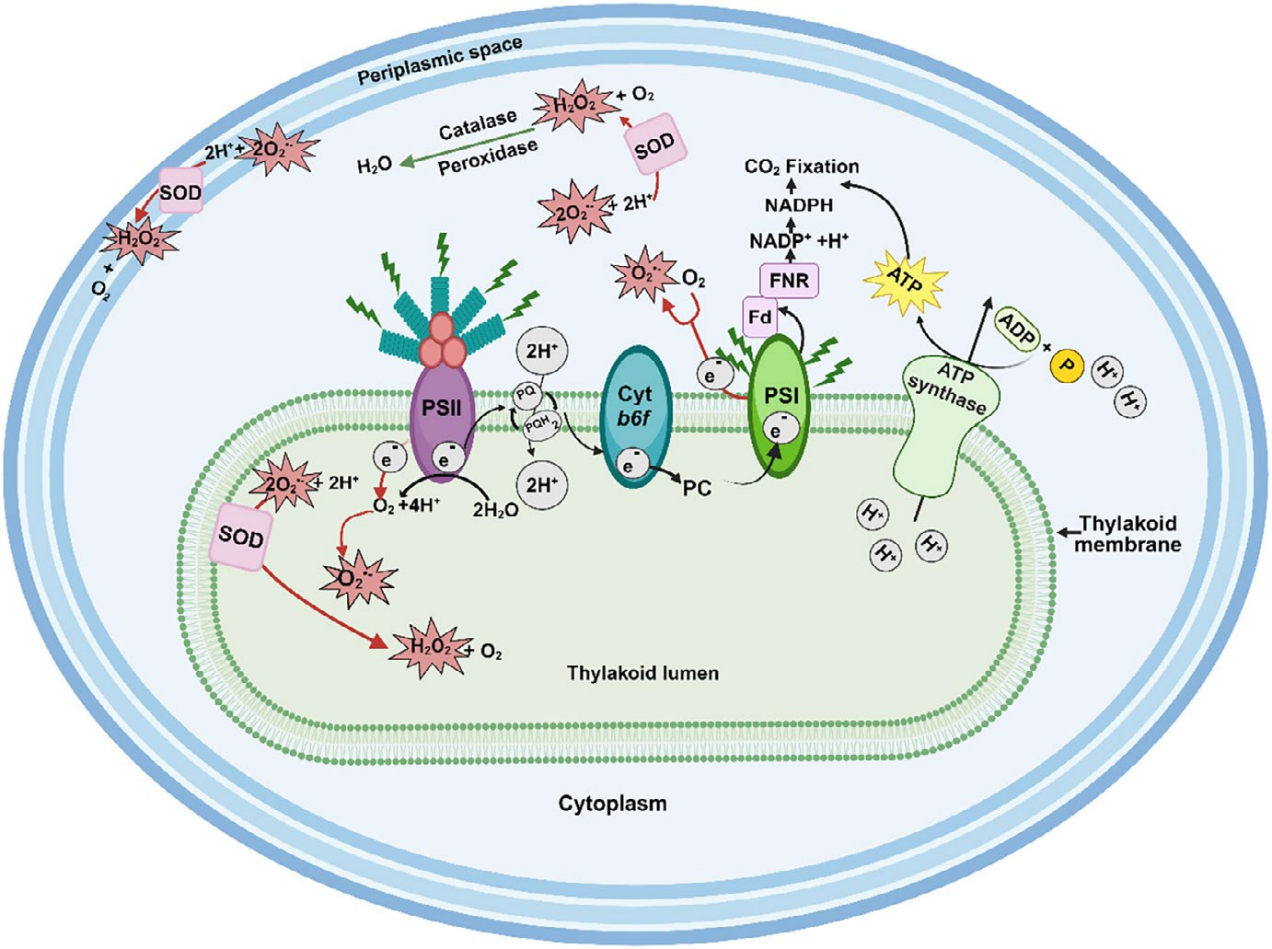
Schematic representation of oxygenic photosynthesis and the generation of superoxide under light conditions inside a cyanobacterial cell. The thylakoid membrane contains photosystem II (PSII) connected to phycobilisomes, photosystem I (PSI), cytochrome *b6f* (cyt *b6f*) and electron transporters plastoquinone (PQ) and plastocyanin (PC). Phycobilisomes capture light energy and transfer it to PSII, resulting in the hydrolysis of water and transfer of electrons to PQ, with the concomitant generation of molecular oxygen (O_2_) and protons (H^+^). PQ then transfers the electrons to PSI via cyt *b6f* and PC, thereby translocating two protons (2H^+^) across the thylakoid membrane into the lumen. The resulting proton gradient powers the synthesis of adenosine triphosphate (ATP) via ATP synthase. PSI transfers electrons to ferredoxin (Fd), which in turn transfers electrons to ferredoxin NADP+ oxidoreductase (FNR) to generate reduced nicotinamide adenine dinucleotide phosphate (NADPH). Both NADPH and ATP are used to power cellular processes, such as CO_2_ fixation (Hagemann et al. 2016). Increased light exposure may result in excess electrons being fed into the electron transport chain, resulting in the generation of superoxide (O_2_^•−^) (Latifi, Ruiz, and Zhang 2009). Dismutation of O_2_^•−^ to hydrogen peroxide (H_2_O_2_) potentially occurs in different species via superoxide dimutases (SODs), in the cytoplasm, thylakoid lumen or periplasmic space (Herbert et al. 1992; Li et al. 2002; Napoli et al. 2021; Raghavan, Rajaram, and Apte 2013, 2015). The image was generated in BioRender©.

Reactive oxygen species (ROS) including singlet oxygen (^1^O_2_), superoxide (O_2_^•−^), hydroxyl radicals (OH^•^) and hydrogen peroxide (H_2_O_2_) can be generated in cyanobacteria during the course of aerobic respiration (Stebegg et al. 2023; Soo et al. 2017) and oxygenic photosynthesis, which occur on both the cell and thylakoid membranes in most species (Hamilton 2019; Hakkila et al. 2014; Latifi, Ruiz, and Zhang 2009) (Figure 1). For example, under high light conditions, the triple state chlorophyll in photosystem II reacts with O_2_ to generate O_2_^•−^ (Figure 1). Similar byproducts are created during photorespiration in the cytoplasm as a result of the light‐dependent use of O_2_ to recapture organic carbon lost through Rubisco's oxygenase reaction (Hagemann et al. 2013; 2016; Hamilton 2019). Because O_2_^•−^, cannot diffuse through membranes, it rapidly oxidises iron sulphur clusters in enzymes, deactivating them and releasing Fe^2+^. No enzymatic pathways exist to remove OH^•^ or ^1^O_2_ (Rachedi, Foglino, and Latifi 2020). OH^•^ is highly reactive with organic molecules and rapidly becomes diffusion limited (Latifi, Ruiz, and Zhang 2009; Rachedi, Foglino, and Latifi 2020). ^1^O_2_ is inactivated non‐enzymatically by carotenoids (Schäfer, Vioque, and Sandmann 2005) and α‐tocopherol (Inoue et al. 2011).

In order to survive in an increasingly oxygenated environment, organisms had to evolve enzymes to reduce cellular damage from superoxide (O_2_^•−^) (Case 2017; Hamilton 2019; Ślesak et al. 2019, 2016). Superoxide dismutases (SODs) and superoxide reductases (SORs) both reduce superoxide, but SODs are the only autonomous enzymes known to date that can disproportionate O_2_^−•^ to O_2_ and H_2_O_2_, which are further converted to water and O_2_ by catalases (CAT) or to water by thiol‐, NADH‐ or cytochrome‐dependent peroxidases, thereby enabling cells to maintain their intracellular homeostasis (Case 2017; Johnson and Hug 2019). Molecular clock dating and phylogenetic studies of SODs suggest that the last common ancestor of crown group cyanobacteria may already have been equipped with essential detoxification enzymes (Boden et al. 2021; Johnson and Hug 2019; Ślesak et al. 2016).

Four different isoforms of SODs exist based on their metal co‐factors, namely, CuZnSODs containing copper and zinc, FeSODs containing iron, MnSODs with manganese and NiSODs that has nickel in its active site. Sequence similarity and structure make it difficult to differentiate between MnSODs and FeSODs, suggesting that they have a common ancestor (Harada et al. 2021; Johnson and Hug 2019). However, differentiation of FeSODs and MnSODs is potentially possible based on unique amino acid motifs (Priya et al. 2007). Reduction of Mn^3+^ to Mn^2+^, with its higher midpoint reduction potential, is energetically preferable to the reduction of Fe^3+^ to Fe^2+^, explaining the lower Fenton reactivity of Mn^2+^ and making MnSODs more stable under conditions of oxidative stress (Miller 2012). Together, MnSODs/FeSODs are the most widely spread SODs within cyanobacteria, with CuZnSODs more rarely encountered (Boden et al. 2021; Harada et al. 2021). NiSODs occur primarily in saltwater strains, mainly in the more recently evolved picocyanobacteria (Boden et al. 2021; Dupont et al. 2008; Harada et al. 2021). Molecular dating of the four SOD isoforms into the cyanobacterial genomic tree indicates that CuZnSODs were present in the cyanobacterial lineage prior to the GOE after the cyanobacteria had diverged from their non‐photosynthetic relatives, the Vampirovibrionia (Boden et al. 2021). The genes encoding MnSODs (*sodA*)/FeSODs (*sodB*) made an appearance after the GOE spread into new lineages throughout the Proterozoic, while NiSODs (*sodN*) appeared during the Proterozoic when cyanobacteria moved into the open ocean (Boden et al. 2021).

The distribution of SODs within different subcellular compartments of cyanobacteria is dependent on the source of O_2_^•−^ (Figure 1). *Spirulina platensis* expresses a cytosolic FeSOD that is induced under increased salinity and iron availability (Ismaiel et al. 2014). Photosynthesis during the light phase results in the upregulation of *sodB* expression, accompanied by an increase in FeSOD activity in the cytoplasm of *Synechocystis* sp. PCC6803 cultures under normal oxic conditions (Kim and Suh 2005). Attempts to complement a *Synechocystis* sp. PCC6803 *sodB* deletion mutant with *sodC* from *Synechococcus* sp. CC9311 revealed that the CuZnSOD (encoded by *sodC*) was located in the thylakoid membrane and lumen and was unable to replace FeSOD activity in the cytoplasm (Ke et al. 2014). MnSOD can also be located in the membrane, as observed in *Synechococcus* sp. PCC7942 (Herbert et al. 1992) and *Nostoc* sp. PCC7120 (Li et al. 2002). Therefore, SODs can be directed to membranes or pass through into compartments (thylakoid or periplasmic space for cyanobacteria) based on the signal peptide encoded on the genome (Russo and Zedler 2021). The analysis of the leader peptide for MnSOD from *Nostoc* sp. PCC7120, combined with overexpression studies, indicated that several forms of MnSODs were encoded by a single *sodA* gene. These include a full length membrane‐bound protein and functional truncated proteins that were located in both the cytoplasm or membrane fractions (Raghavan, Rajaram, and Apte 2013, 2015). FeSOD production increased 6–8‐fold during the transition from nitrogen replete to nitrogen depleted conditions in *Nostoc* sp. PCC7120, while MnSOD was principally found on the luminal side of the thylakoid membrane. It was proposed that *Nostoc* sp. PCC7120 could modulate the proteolytic processing of the N‐terminal signal and linker peptides of membrane‐targeted MnSOD in response to nitrogen availability (Raghavan, Rajaram, and Apte 2015).

Export of MnSODs to the thylakoid lumen or periplasmic space is supported by the sequence analysis of one of the two MnSODs encoded by *Chroococcidiopsis* sp. CCMEE 029. A signal peptide for the TAT signal transduction system was identified for one of the MnSODs (SodA2.1), suggesting that it was localised in the periplasmic space and/or the thylakoid lumen (Napoli et al. 2021). The second MnSOD (SodA2.2) and the CuZnSOD (SodC) carried no signal peptide and were presumably present in the cytoplasm. The expression levels of all three SOD genes were significantly elevated after 60 min desiccation in the dark (Napoli et al. 2021). In summary, the type, number and exact location of SOD isoforms may depend on the environment, species evolution and functionality. Yet, no research on SOD gene expression and activity in early‐branching cyanobacteria, such as *Pseudanabaena* spp., has been conducted.

Molecular clock analyses have identified some deep branching clades of cyanobacterial species that can be traced back to the late Archean, prior to the GOE (Boden et al. 2021; Jahodářová et al. 2018; Sánchez‐Baracaldo 2015; Sánchez‐Baracaldo et al. 2017). It includes several *Pseudanabaena* spp., and net O_2_ production rates by one of these early branching marine strains, *Pseudanabaena* sp. PCC7367, are significantly higher in cultures grown anoxically at 0.2% atmospheric CO_2_ (Herrmann et al. 2021). Control cultures grown under present‐day levels of CO_2_ and O_2_ or controls supplemented to 0.2% CO_2_ demonstrated significantly lower rates of O_2_ release per chlorophyll a (Chl a) content. This raises the question as to the potential of host SODs inactivating the potentially increased levels of O_2_^•−^ in cyanobacteria growing in a simulated oxygen‐free, early Earth atmosphere. Furthermore, this study tried to elucidate whether the growth of *Pseudanabaena* sp. PCC7367 correlated to atmospheric pO_2_ and cellular SOD activity. *Pseudanabena* sp. PCC7367 encodes three putative SODs, namely, MnSODs (*sodA*), FeSODs (*sodB*) and CuZnSODs (*sodC*) (Boden et al. 2021; Harada et al. 2021). This study investigates the expression and activity of these SODs over 24 h under a simulated high‐CO_2_, anoxic atmosphere to assess the role of atmospheric O_2_ in cyanobacterial growth. Altogether, our data provide insights into diurnal expression and activity of SODs in a deep‐branching cyanobacterium under an anoxic, early Earth simulation compared to present‐day oxygen‐rich conditions.

## 2 Materials and Methods

### 2.1 Culture Conditions

*Pseudanabaena* sp. PCC7367 was purchased from the Pasteur Culture Collection (Paris, France) and maintained under normal present atmospheric levels (PAL) of 0.04% CO_2_ (low CO_2_—LC), under photosynthetically active photon flux (PPFD) of 20 μmol photons m^−2^. s^−1^, 65% humidity and a (16:8) light/dark cycle in an artificial salt medium, ASNIII (Herrmann et al. 2021) (25). The experimental cultures used in this study were similarly maintained for 6 months under their respective atmospheric conditions of PAL (LC) or PAL supplemented to 0.2% CO_2_ (HC) in a Percival E22 growth chamber (CLF Plant Climatics, Germany) illuminated with Radium NL 18 W/840 Spectralux Plus white light bulbs (Germany). Similarly, cultures were grown under anoxic Archean simulated conditions in N_2_ gas containing 0.2% CO_2_ (Archean) in an anaerobic workstation (MEGA‐4, GS Glovebox, Germany) fitted with Cree CXB1816 LED lights (USA) (Herrmann et al. 2021; Enzingmüller‐Bleyl et al. 2022). The 0.2% CO_2_ fell at the lower end of the predicted spread of Archean atmospheric CO_2_ levels (Catling and Zahnle 2020). We ensured the light spectra and light intensities were the same under all three growth conditions, using an optical multichannel analyser (USB2000^+^ Ocean Optics, Germany).

Comparative growth curves of acclimated cultures were initiated at an initial Chl a concentration of 0.04 μg. mL^−1^ in 500 mL of the ASNIII medium, in triplicate, in large Fernbach flasks for each of the three atmospheric conditions. The Chl a content was measured every second workday, with samples for the assessment of cellular carotenoid, protein and glycogen content collected in parallel, for a total of 28 days. For SOD transcriptional and activity analyses, cells were harvested at a similar Chl a concentration (~2 μg. mL^−1^) during the late exponential phase to maximise RNA and protein yields, on days 8–9 under Archean atmospheric conditions, days 12–13 for the HC and days 13–14 for the LC culture conditions.

The total number of cells per milliliter was determined using a Neubauer counting chamber and used to calculate cellular SOD activity. The cell count was performed for each biological triplicate on day 9 for the cultures grown under an Archean simulated atmosphere, day 12 for HC and day 13 for LC grown cultures, the same day on which the protein and RNA samples were taken.

### 2.2 Chl a and Carotenoid Extraction

A 2 mL volume of culture was collected in 2 mL brown tubes and centrifuged (5 min, 10,000 RCF, Hermle LaborTechnik GmbH—Z 233 M‐2 Microliter Centrifuge), and the cell pellet was drained. Around 100 μg (a small spatula full) of 0.1 mm silica beads (BioSpec, USA) and 1.5 mL of neutralised 90% MeOH were added to the drained pellet, followed by disruption (FastPrep bead beater FP 120, Thermo Electron Corporation) at 6.5 m. sec^−1^ speed for 45 s (twice) and incubation overnight in the dark at 4°C. The next day, the lysate was vortexed and centrifuged (15 min at 10,000 RCF), and the absorbance of the supernatant was measured at 665 nm (Chl a) and 470 nm (carotenoid), using a spectrophotometer (Hellma, Agilent 8453, China). Chl a (Meeks and Castenholz 1971) and carotenoid (Wellburn 1994) content was calculated (Herrmann et al. 2021). The growth rate was determined from the Chl a growth curve from day 0 to day 12.

### 2.3 Protein and Glycogen Quantification

The protein and glycogen contents of cells provide insights into the growth of the culture and its nitrogen and carbohydrate resources (Herrmann and Gehringer 2019; Klotz et al. 2016). A cell lysate was prepared from 2 mL of pelleted culture material (12,000 RCF for 5 min) that was resuspended in 1 mL of lysis buffer (5 mM NH_4_SO_4_, 2 mM DTT, 1 mM MgCl_2_, 20 mM KH_2_PO_4_ (pH 8.5)) with ~100 μg (a small spatula full) of 0.1 mm silica beads (BioSpec, USA), followed by mechanic disruption as above. The samples were additionally freeze/thawed in liquid nitrogen before and after each round of bead beating. To pellet cell debris, the cell extracts were centrifuged at 13,000 RCF for 15 min (HERMLE, Z233 M‐2, Germany), and the supernatant was transferred to a fresh 1.5 mL tube and stored at −20°C. These cell lysates were used for both protein and glycogen determinations.

The protein content of the biomass was assessed using the Bradford assay. A 1:2 serial dilution series of an Albumin Fraction V (Sigma‐Aldrich, USA) stock solution of 2 mg. mL^−1^ was made ranging from 0.488 μg. mL^−1^ to 500 μg. mL^−1^ in lysis buffer. For protein determination, a 50 μL volume of blank, standard dilution or sample was added into the wells of a 96‐well clear bottom plate, and 250 μL of the Bradford reagent (Merck, Darmstadt, Germany) was added. The plate was incubated at room temperature for 10 min after which the absorbance at 595 nm was determined (Multiskan FC, Thermo Fisher Scientific). Experimental protein concentrations were read off the standard curve (Herrmann and Gehringer 2019). *R* ^2^ values ranged between 0.95 and 0.99.

In order to measure the glycogen content of the cells, a 1:2 serial dilution series of a stock solution of oyster glycogen (Sigma, Germany) of 2 mg. mL^−1^ was made ranging from 0.488 μg.mL^−1^ to 500 μg.mL^−1^ in the lysis buffer. A volume of 200 μL of standard dilution, sample or blank was transferred into a 2 mL reaction tube, and a volume of 500 μL of the ice‐cold anthrone (Sigma‐Aldrich, Germany) reagent (2% w/v in 98% sulphuric acid) was added and then incubated for 30 min at 80°C. Afterwards, 250 μL of each sample was transferred into individual wells of a clear bottom 96‐well plate, and the absorbance was read at 620 nm (Multiskan FC, Thermo Fisher Scientific). Experimental glycogen concentrations were calculated off the standard curve (Herrmann and Gehringer 2019). *R* ^2^ values ranged between 0.98 and 0.99.

### 2.4 Quantification of Oxygen Medium Levels

Oxygen accumulation both within and outside the cell influences SOD expression levels (Kim and Suh 2005). Oxygen levels in stationary cultures were measured over 24 h to determine the time points for assessing expression and activity of SOD in *Pseudanabaena* sp. PCC7367 grown under the three different atmospheres investigated. The O_2_ levels were initially recorded in cultures without agitation to mimic the proposed shallow water marine oxygen oases identified in the Archean fossil record (Catling and Zahnle 2020; Riding, Fralick, and Liang 2014). Additionally, the cultures were stirred to ensure maximal O_2_ release from the medium during the dark phase and to identify a time point with the lowest possible SOD activity. Levels of dissolved O_2_ (μM. L^−1^) in the culture media of *Pseudanabaena* sp. PCC 7367 (*n* = 3) were measured (Robust Oxygen Probes OXROB10, attached to the Firesting‐O2, Pyroscience, Germany) over a full diurnal cycle under each atmospheric condition investigated. Cultures were then gently stirred, and the dissolved O_2_ levels were again recorded.

### 2.5 SOD Protein Characterisation and Structural Prediction of *Pseudanabaena* sp. PCC7367

The three SODs encoded within *Pseudanabena* sp. PCC7367 (Boden et al. 2021; Harada et al. 2021) were investigated with respect to their potential membrane binding and signalling domains. To identify whether transmembrane domains existed and where, the amino acid sequences of each SOD were subjected to DeepTMHMM (https://dtu.biolib.com/DeepTMHMM Hallgren et al. 2022).

The 3D models of the SODs encoded by *Pseudanabaena* sp. PCC7367 (Boden et al. 2021; Harada et al. 2021) were generated on the Phyre2 model server (Kelley et al. 2015) and visualised using the Swiss‐PDB viewer (http://www.expasy.org/spdbv/) (Guex, Peitsch, and Schwede 2009). Alphafold 2 (Jumper et al. 2021; Varadi et al. 2021) was used to generate the protein structures of the CuZnSODs, MnSODs and FeSODs of *Pseudanabaena* sp. 7367. Final images were coloured for confidence in the secondary structure predictions using PyMOL 2.3 (DeLano 2020).

### 2.6 Genetic Potential of SOD‐Associated Genes in *Pseudanabaena* sp. PCC7367

The KEGG Database (Kanehisa et al. 2022) was searched for annotated SORs, peroxidases/peroxiredoxins and catalase genes encoded on the *Pseudanabaena* sp. PCC7367 genome (Shih et al. 2013). As no catalase or SORs were annotated in KEGG, the genome of *Pseudanabaena* sp. PCC 7367 (NC_019701.1) was additionally screened, using tBLASTn (Altschul 1991), for the presence of 1Fe‐SOR from the archaea *Pyrococcus furiosus* DSM 3638 (PF1281) and 2Fe‐SOR from the bacteria *Desulfovibrio vulgaris* DP4 (Dvul_0204) (Sheng et al. 2014), obtained from the Superoxide Reductase Gene Ontology Database (SORGOdp) (Lucchetti‐Miganeh et al. 2011), as well as the catalase gene from *E. coli* K12 MG1655 (YP_025308). Furthermore, to ensure the complementary metal cofactors were accessible for SOD isoform activities, the genome of *Pseudanabaena* sp. PCC7367 was screened for the presence of Mn(II) transporters (MntABC) (Bartsevich and Pakrasi 1995), Zn(II) transporters (ZnuABC) and Cu(II) transporters (CtaA and PacS) (Sharon et al. 2014), using the characterised protein sequences from *Synechocystis* sp. PCC6803 (NC_000911.1).

### 2.7 Detection of SOD Gene Expression Levels

A set of primers were designed to target the *sodA* (MnSOD), *sodB* (FeSOD) and *sodC* (CuZnSOD) genes identified in *Pseudanabaena* sp. PCC7367 (Table S1), as well as the housekeeping RNA polymerase beta subunit gene, *rpoC1* (Alexova et al. 2011; Enzingmüller‐Bleyl et al. 2022) using NCBI Primer‐BLAST Software (Ye et al. 2012). To avoid self‐complementarity, scores generated by PCR primer inspector (www.molbiotools.com/primerinspector.php) and NetPrimer (www.premierbiosoft.com/netprimer/) were considered. Primers were validated by sequencing of the PCR products as well as determining their binding efficiencies to both genomic DNA (gDNA) and copy DNA (cDNA) generated from total RNA extractions (Table S2).

Samples for RNA extraction were taken in late exponential phase on days 8 and 9 for cultures grown under anoxic conditions, days 12 and 13 for cultures cultured under an HC conditions and days 13 and 14 for LC atmospheric conditions, with Chl a concentrations of ~2 μg. mL^−1^, which had similar protein and glycogen concentrations (Figure S1). Based on the observed dissolved O_2_ levels in stationary cultures (Figure S2), samples were collected 2 h after the lights went on, 2 h before they went off (14 h: at maximum levels of dissolved O_2_ in the medium), 2 h after the lights went out (18 h) and 2 h prior to them being switched on again (22 h). The following evening, an additional sample (19 h) was taken, 3 h after darkness, from the stirred cultures, for the assessment of the baseline SOD expression levels at minimum oxygen levels in the dark.

A 90 mL culture volume was added to 10 mL of ice‐cold stop solution (5% ROTI Aqua‐Phenol (ROTH, Germany) in p.A. ethanol) at each time point. The cells were harvested by centrifugation (12,000 RCF for 10 min, Hermle Z 513 K, Germany), and the pellet was weighed. RNA was extracted using the NucleoSpin RNA Plant Kit (MACHEREY‐NAGEL, Germany) according to the manufacturer's instructions. The initial lysis step was modified to increase the yield of RNA as follows. An approximately 200 mg pellet was resuspended in 350 μL of RA1 solution and then transferred to a sterile, RNAse‐free 2 mL tube containing around 100 μg of 0.1 mm silica beads (BioSpec) and 3.5 μL of β‐mercaptoethanol (ROTH, Germany). The cells were subjected to two cycles of a rapid freeze/thaw lysis step in liquid nitrogen followed by mechanical disruption (FastPrep) for 45 s at 6.5 speed. The cell debris was removed by centrifugation (1 min, 11,000 RCF), and the supernatant was used for further RNA purification following the manufacturer's instructions. RNA was quantified using a NanoDrop Lite Spectrophotometer (Thermo Fisher Scientific), while the quality was checked by agarose gel electrophoreses on a 1% w/v Tris‐acetate‐EDTA (TAE) gel (Figure S4). The purity was determined by PCR using the housekeeping gene primer pair to ensure that no DNA remained. RNA was subjected to repeat DNA digestion if a positive PCR was observed. A 10 μL volume of 10‐fold DNAse I reaction buffer (New England Biolabs, catalogue‐B0303S), 5 units (2.5 μL) of RNase free DNAse I (New England Biolabs, 2000 u. mL^−1^) and 100 μL of nuclease‐free water were added for every 10 μg of RNA, and incubated for 30 min at 37°C. After DNAse treatment, the quantity and quality of RNA were again checked as previously described. Complementary DNA (cDNA) was reverse transcribed from ~1 μg of high‐quality RNA using the ProtoScript II First Strand cDNA Synthesis Kit (New England Biolabs, Germany). Newly synthesised cDNA was purified using the NucleoSpin Gel and PCR Clean‐up kit (Germany) and quantified using a NanoDrop Lite Spectrophotometer. A test PCR using the control primer pair was conducted to confirm successful cDNA synthesis.

Expression of each gene ie: *sodA, sodB, sodC* and *rpoC1* was assessed using the 2x iTaq Universal SYBR Green Supermix (Bio‐Rad, USA) and the respective primer pairs. An 8 μL volume of the master mix containing 1 μM of forward and reverse primers was aliquoted into the 96‐well PCR plate (STARLAB, Germany) for each reaction, and 10 ng of cDNA in 2 μL was added. For no template controls (NTCs), 2 μL of ddH_2_O was added instead of the template. The reaction volumes were mixed by pipetting, and the plate was sealed with plastic adhesive foil (Bio‐Budget, Germany) and labelled. Three technical replicates were conducted for each reaction on two different days with an initial incubation of 10 min at 50°C to activate the polymerase, followed by a single denaturing step of 5 min at 95°C and then 40 cycles of 10 s at 95°C, 20 s at 55°C and 10 s at 72°C, and a final elongation step of 5 min at 72°C. The relative expression of the SOD genes relative to the *rpoC1* housekeeping gene was calculated as described in the Supplementary Text.

### 2.8 Assessment of SOD Activity

The protein was extracted from *Pseudanabaena* sp. PCC 7367 cultures during the late exponential phase at the same time points and similar Chl *a* concentration as the transcriptional analysis (Figure S1). The cell pellet from a 50 mL culture volume was obtained by centrifugation at 8,000 RCF for 10 min (Hermle Z 513 K, Germany). The pellet was resuspended in 2 mL of freshly prepared lysis buffer (5 mM NH_4_SO_4_, 2 mM DTT, 1 mM MgCl_2_, 20 mM KH_2_PO_4_ (pH 8.5)) and sonicated on ice for six cycles of 1 min, with a break between every minute at 4°C, 130 W, 20 kHz and 50% amplitude using an ultrasonicate homogeniser (UV 220, Bandelin, Germany). The sonicated extract was centrifuged (Sorvall LYNX 6000, Thermo Fisher, Germany) at 4,3185 RCF at 4°C (60 min). The supernatant was carefully transferred into a new 2 mL tube and kept at 4°C before performing the colorimetric SOD enzyme activity assay. The concentration of the extracted protein was measured using the Bradford assay as previously described.

The total SOD activity of soluble cellular proteins was determined using a microtiter plate enzyme inhibition assay (Peskin and Winterbourn 2017). The assay is based on the reduction of the water‐soluble tetrazolium salt (WST‐1) with a superoxide anion, producing a water‐soluble formazan dye that can be calorimetrically quantified by measuring the absorbance between 410 and 450 nm. The superoxide anion is produced through the oxidation of hypoxanthine by xanthine oxidase. SOD inhibits the formation of formazan by catalysing the dismutation of the superoxide anions into hydrogen peroxide and molecular oxygen, thereby decreasing the reduction of WST‐1 and hence the absorbance. An assay buffer was prepared with 100 mM sodium phosphate (pH 8.0), 0.1 mM diethylenetriaminepentaacetic acid (Sigma‐Aldrich, Germany) and 0.1 mM hypoxanthine (Sigma‐Aldrich, Germany). A stock solution of 10 mM hypoxanthine (Sigma‐Aldrich) solution was prepared in dimethyl sulfoxide (DMSO, ROTH, Germany) and then diluted as required. 10 mL of a 10 mM solution of WST‐1 (Dojindo Molecular Technologies, USA) was prepared and wrapped in aluminium foil to protect it from light. A 2 mg. mL^−1^ solution of catalase (Sigma‐Aldrich) was dissolved in phosphate buffer pH 7 and stored at 4°C. Bovine CuZnSOD (Sigma‐Aldrich) was used to generate a standard curve or relative SOD activity as follows: a (1:2) dilution series of bovine CuZnSOD was prepared ranging from 0.0488 μg. mL^−1^ to 100 μg. mL^−1^ in lysis buffer. A 1 μL volume of blank, standard dilution or sample was added to wells of a 96‐well plate, in duplicate, at 24°C. Afterwards, 0.2 mL of assay buffer containing sufficient xanthinin oxidase (Peskin and Winterbourn 2017) was added to each well. The 96‐well plate was immediately placed into the plate reader (Infinite f200 PRO, TECAN) and shaken for 5 s, after which the absorbance was measured at 415 nm for 5 min. The amount of CuZnSOD added in the assay (X) was plotted against the calculated percentage of the inhibition of WST‐1 reduction (Y), from which the amount of activity relative to bovine CuZnSOD were determined. The *R* ^2^ values fell between 0.96 and 0.98 (Figure S5).

### 2.9 Statistics

Statistics was carried out using tools available in SigmaPlot 13.0 (Systat Software Inc.). The datasets were tested for the homogeneity of variance, using Levene's test, and for normality, the Kolmogorov–Smirnov test was used. Differences in the growth associated variables of chlorophyll a, carotenoid, protein and glycogen content, as well as media O_2_ concentration were assessed using repeated measure One Way Analysis of Variance/on ranks, followed by Tukey’s honest significant difference (HSD)/ Holm‐Sidak method as post‐hoc test.

A two‐way repeated measures ANOVA (two‐factor repetition) with treatment and time as factors for parameters sampled during the 24 h sampling cycle was applied, which included SOD gene expression levels (*sodA* (MnSOD), *sodB* (FeSOD), *sodC* (CuZnSOD) and total SOD expression) and SOD enzyme activity, as well as the concentration of dissolved O_2_. In case of significant differences in parameters caused by environmental conditions, Duncan's method was used as a post hoc test to identify diverging groups. Moreover, Pearson's correlation was then conducted to identify the relationship between variables of 24 h sampling.

## 3 Results

Comparative growth curves were set up under the three defined atmospheric conditions to assess which best supported *Pseudanabaena* sp. PCC7367 growth. This permitted the identification of comparative sampling time points for quantification of SOD transcription and activity over a full diurnal cycle.

### 3.1 *Pseudanabaena* sp. PCC7367 Exhibits a Higher Growth Rate, Accompanied by Increased Glycogen and Protein Levels Under Anoxic Growth Conditions

**FIGURE 2:**
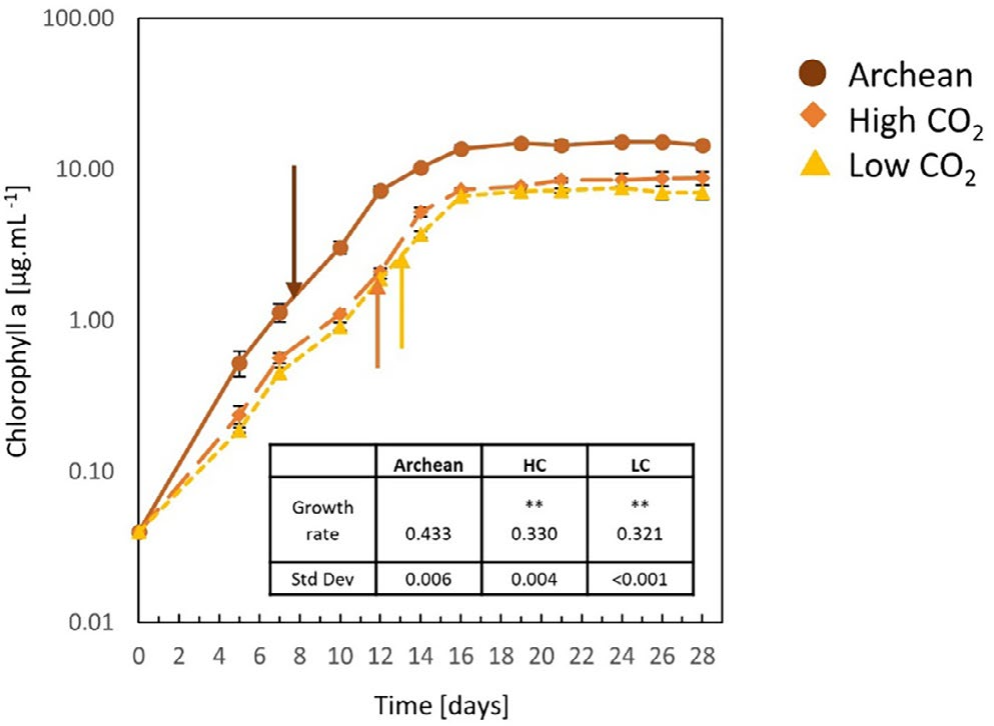
Growth assessment of *Pseudanabaena* sp. PCC7367 under three atmospheric conditions. Triplicate cultures of *Pseudanabena* sp. PCC7367 were inoculated at 0.04 μg mL^−1^ Chl a, monitored for 28 days for Chl a content and used to calculate the growth rate (inset table) for days 0–12. Samples were collected at similar Chl a content from unstirred cultures for RNA extraction and enzyme activity determinations on days 8 and 9 under Archean atmospheric conditions (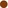), days 12 and 13 for the high CO_2_ atmosphere grown cultures (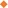) and days 13 and 14 for present‐day atmospheric conditions (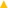), indicated by arrows. Bars represent the standard deviation (*n* = 3). **p < 0.001 for the increased growth rate under Archean conditions compared to HC and LC (pairwise multiple comparison, Holm–Sidak method; Table S6).

Growth rates were determined from the Chl a growth curves generated for *Pseudanabaena* sp. PCC 7367 grown under the Archean simulated anoxic atmosphere and the HC and LC oxygen‐rich atmospheric conditions (Figure 2; Table S6). *Pseudanabaena* sp. PCC 7367, grown under an anoxic atmosphere, exhibited a significantly higher growth rate compared to those cultures grown under present‐day atmospheric oxygen levels at LC (*p* < 0.001) and HC (*p* < 0.001; Figure 2; Table S6). Carotenoid levels were also significantly higher in anoxic grown cultures, compared to cultures grown under LC (*p* = 0.001) conditions (Figure S1A; Table S6). However, the Chl a/carotenoid ratio was not significantly different between the cultures on the days of sampling, nor overall (*p* = 0.219; Table S6). Glycogen levels were significantly raised under anoxic growth conditions (Figure S1B; Table S6) compared to LC (*p* = 0.004) and HC (*p* = 0.035) atmospheres (Tukey's test: all pairwise multiple comparison procedures). Protein levels were significantly higher in the culture material from Archean grown cultures than those in biomass from LC (*p* = 0.002) or HC (*p* = 0.029) growth conditions (Figure S1C; Table S6). In summary, *Pseudanabaena* PCC7367 grown under anoxic conditions had a significantly higher growth rate than cultures grown under oxic conditions. The significantly higher levels of glycogen and protein accumulation in stationary phase cultures point to increased cell vitality, permitting the accumulation of excess carbon while maintaining the carbon‐to‐nitrogen balance.

### 3.2 Medium O_2_ Levels Remain Similar During Oxygenic Photosynthesis; However, Dark Phase Levels are Significantly Reduced Under Anoxic Conditions

**FIGURE 3:**
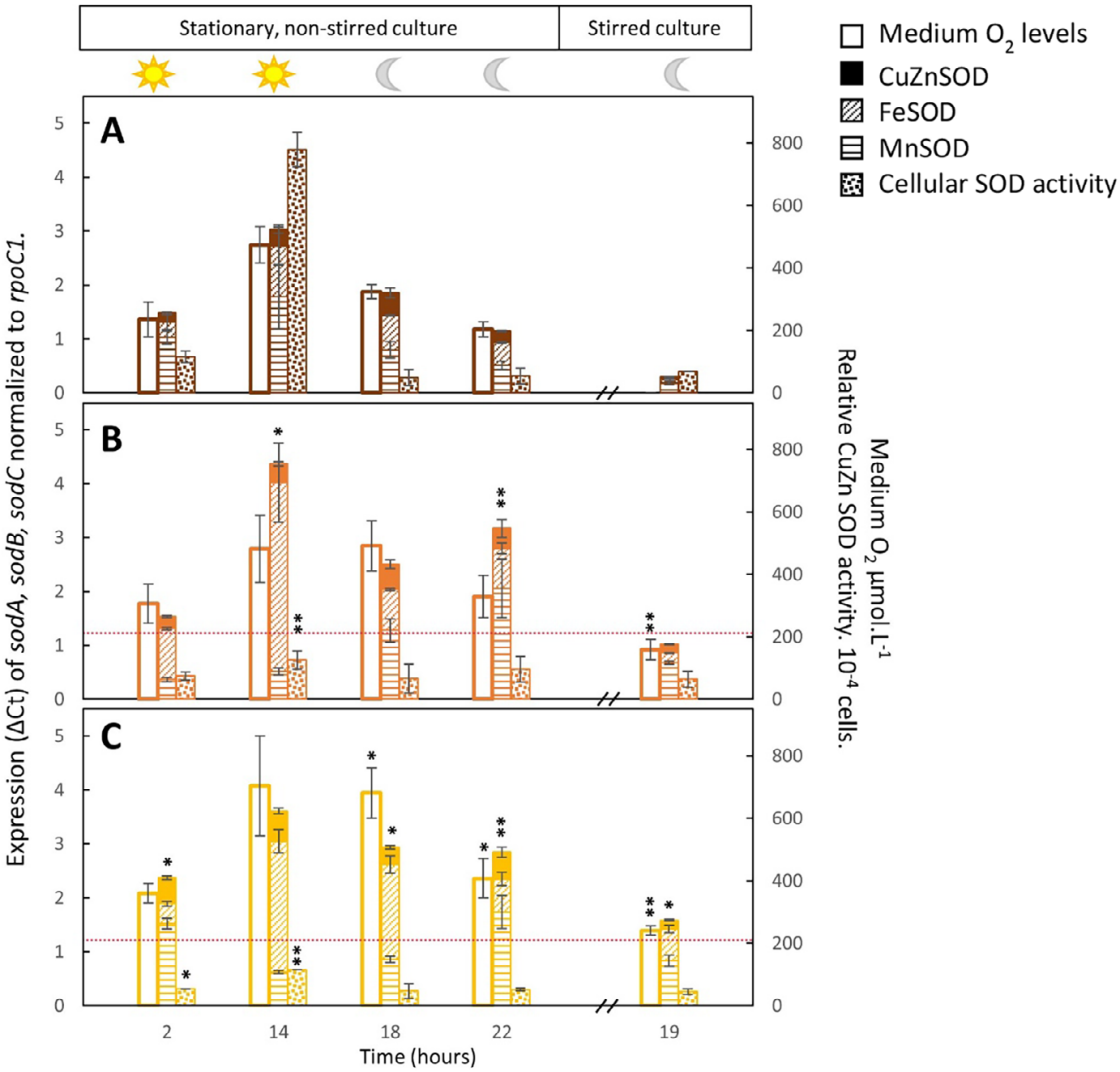
Expression and activity of cellular superoxide dismutase against medium O_2_ concentration. The expression of the individual genes, namely *sodA*, encoding a putative MnSOD (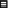), *sodB*, encoding FeSOD (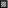) and *sodC*, encoding a putative CuZnSOD (■) is plotted with the concentration of O_2_ recorded in the medium (□) and the relative cellular SOD activity (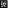) for conditions simulating the anoxic Archean atmosphere (A) and present‐day oxic conditions with (B) or without (C) CO_2_ supplementation. Data are presented for the first four sampling time points, namely, 2 h after the lights went on (2 h), 2 h before they went off (14 h), 2 h after they went off (18 h) and 2 h before the lights went on again (22 h). The cultures were subsequently stirred, and the last sample was obtained at 19 h in the dark to obtain a reading equilibrated with the atmosphere, not influenced by photosynthesis. Bars represent the average of three biological replicates and their standard deviation. The dotted red line indicates the dissolved O_2_ levels of 206 μmol.L^−1^ in the artificial seawater medium. The O_2_ concentration was below the detection limit in the Archean simulation experiments at sample point 19. Significant differences of LC and HC to Archean parameters are provided with * for *p* < 0.05 and ** for *p* ≤ 0.001 (Table S8).

Given that O_2_ accumulation both within and outside the cell influences SOD expression levels (Kim and Suh 2005) and as *Pseudanabaena* PCC7367 releases significantly more O_2_ under anoxic conditions (Herrmann et al. 2021), the residual O_2_ levels in the medium was tracked over a full day cycle. To obtain basal levels of O_2_ retention in agitated cultures, the O_2_ levels in gently stirred cultures were measured for an additional 24 h. Oxygen levels in the medium were consistently higher during the period of active photosynthesis than during the dark phase for all conditions measured (Figure S2; Table S7). Standard deviations of triplicate measurements were large for stationary cultures, illustrating the influence of O_2_ bubble attachment to the sensors or clumping of culture mass on or near the sensor. Stirred cultures had less variation between triplicate measurements, reflected in smaller standard deviations (Table S7). Medium O_2_ levels revealed no significant differences between cultures grown under anoxic or oxygen rich conditions during the day (Figure 3) at time points 2 and 14 h. A significant reduction in medium O_2_ levels was recorded for stationary cultures grown under the Archean anoxic simulation compared to LC culture conditions during the dark phase at 18 h (*p* = 0.012) and 22 h (*p* = 0.04) (Table S7). Moreover, stirred cultures of the Archean anoxic simulation were significantly reduced in O_2_ levels during the dark phase under anoxic conditions compared to LC (*p* < 0.001) and HC (*p* = 0.001) grown cultures (Table S7).

### 3.3 Transcription of *sod* Genes (s*odA* , *sodB* and *sodC* ) Exhibits Significant Increases Under Oxic Growth Conditions not Reflected in Increased Cellular Activities

To determine whether atmospheric oxygen levels influenced the transcription of *sodA, sodB* and *sodC* genes, their expression levels were assessed over 24 h. The genome of *Pseudanabaena* sp. PCC7367 (NC_019701.1) includes three SODs, namely, 696 bp *sodC* (Pse7367_0398) encoding CuZnSOD, *sodB* (PSE7367_RS14055) of 600 bp for FeSOD and a 765 bp gene, *sodA* (Pse7367_0596), for MnSOD (Boden et al. 2021; Harada et al. 2021). Primers to each gene were validated (Tables S1 and S2) and used to quantify SOD gene expression over 24 h in non‐stirred cultures of *Pseudanabaena* sp. PCC7367 grown under the three atmospheres of the anoxic Archean and oxic LC and HC conditions. Additional samples of stirred cultures were collected during the dark phase, representing the lowest levels of O_2_ in the medium and hence the potentially lowest O_2_^−•^ levels (Figure S2).

The total expression of all three genes, *sodA, sodB* and *sodC*, in *Pseudanabaena* sp. PCC 7367 correlates significantly (≙SOD sum, *R* ^2^ = 0.751, *p* = 2.81 × 10^−9^; Table S9) with the dissolved O_2_ levels in the medium, with the highest expression recorded at 14 h (Figure 3), 2 h before the lights went off, under all three atmospheric conditions investigated. The highest expression of combined *sod* (*sodA*, *sodB* and *sodC*) in relation to the housekeeping gene, *rpoC1*, was observed for *Pseudanabaena* sp. PCC7367 grown under HC and was significantly more (*p* = 0.002; Table S8) than under Archean conditions after 14 h of light (Figure 3A). Transcription of combined *sod* (*sodA*, *sodB* and *sodC*) remained significantly raised at LC conditions during the dark phase at time points 18 h (*p* = 0.014) and 22 h (*p* < 0.001) and HC at 22 h (*p* < 0.001) when compared to dark‐phase transcription levels under the Archean simulated atmosphere.

The expression of the individual *sodB* and *sodC* genes was highly significantly correlated (Table S9) to medium oxygen levels (*R* ^2^ = 0.651, *p* = 2.7 × 10^−7^ and *R* ^2^ = 0.675, *p* = 3.76 × 10^−7^, respectively), with the expression of *sodC* correlating strongly to the expression of *sodA* (*p* < 0.001) and *sodB* (*p* < 0.001). While *sodA* showed no significant correlation of expression to medium oxygen levels (*p* = 0.122), it was the only SOD gene that correlated significantly (*p* = 0.019) to total cellular SOD activity (Table S9). The expression of *sodA* was also significantly higher under Archean conditions at 2 h compared to the HC (*p* < 0.001) and LC (*p* < 0.001) conditions, with LC expression levels also significantly raised compared to HC conditions (*p* < 0.001). The expression of *sodA* was also significantly raised at 14 h (*p* = 0.02) compared to HC conditions and afterwards significantly reduced at 18 h (*p* = 0.03), 22 h (*p* = 0.006) and 19 h (*p* < 0.001), as well as LC conditions at 22 h (*p* = 0.012) and 19 h (*p* < 0.001).

In contrast to *sod* (*sodA*, *sodB* and *sodC*) expression levels, a significant (*p* < 0.001) reduction in cellular SOD activity was recorded under HC and LC conditions at 14 and 2 h (LC only) compared to Archean culture conditions, suggesting impaired enzyme activity high atmospheric O_2_ conditions. While no significant change in cellular SOD activity was observed for samples taken after 19 h agitation between all three atmospheres, the expression of *sod* (*sodA*, *sodB* and *sodC*) was significantly raised (*p* = 0.03) under LC stirred conditions (Figure 2C) compared to the anoxic (Figure 2A) Archean simulation, reflecting the significantly raised levels of dissolved O_2_ in the medium. The lowest transcription of *sod* (*sodA*, *sodB* and *sodC*) was measured in stirred cultures grown anoxically, 3 h after the lights went out (Figure 3A) when no free O_2_ was recorded in the medium.

In summary, the expression of total *sod* (*sodA*, *sodB* and *sodC*) under Archean conditions, as well as the expression of individual *sodB* and *sodC* genes, is significantly reduced (*p* < 0.001) when compared to the modern‐day oxygen‐rich atmosphere. The expression of individual *sodB* and *sodC* genes correlates significantly to medium O_2_ levels, whereas the expression of *sodA* does not. Additionally, cellular SOD activity correlates significantly (*p* = 0.019) to the expression of the MnSOD, *sodA*, but neither to *sodB* nor to *sodC* expression.

### 3.4 Protein Sequence Analysis and Structural Modelling Raise Questions as to Individual SOD Gene Expression and Activity

Given the discrepancies in transcriptional levels of the *sodA*, *sodB* and *sodC* genes in *Pseudanabaena* sp. PCC7367 and their cellular activities, the analysis of the signal peptide targeting sequences of the SOD proteins was undertaken. The goal was to ascertain whether SODs could be exported from the cytoplasm to be inserted in the thylakoid or cell membranes or targeted to the thylakoid lumen or periplasmic space. H_2_O_2_ can be actively or passively transported across cellular membranes, but O_2_^−•^ is unable to cross lipid membranes (Hansel and Diaz 2021). Targeting an SOD to the lumen of the thylakoid, to the site of O_2_^−•^ generation, may offer protection from superoxide‐induced damage.

**FIGURE 4:**
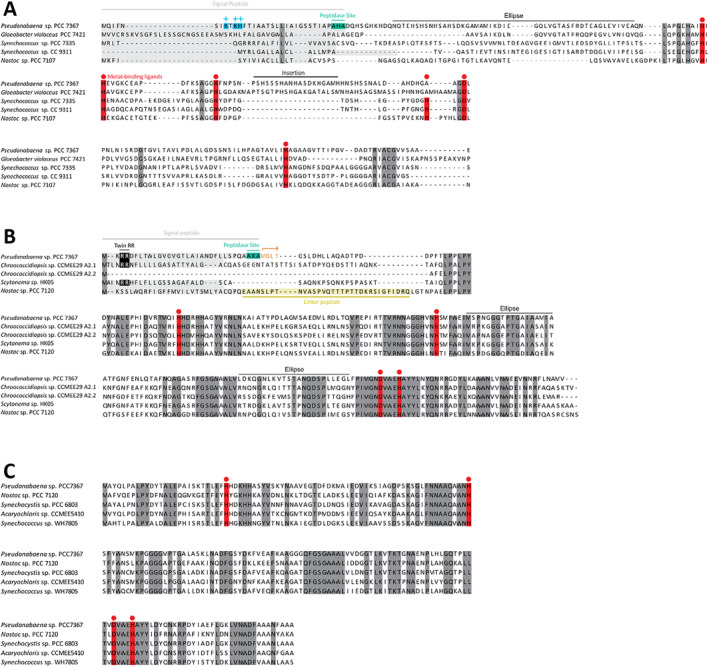
Multiple sequence alignments of the superoxide dismutases encoded on the genome of *Pseudanabaena* sp. PCC7367. (A) The CuZnSOD protein sequence has a positively charged N‐terminal sequence followed by a potential transmembrane segment and AxA signal peptidase cleavage site, which suggests export from the cytosol via the Sec secretory system. (B) The N‐terminus of the *Pseudanabaena* MnSOD carries a potential twin arginine signal (twin RR), transmembrane segment and signal peptidase cleavage site (AxA). The second methionine residue following the cleavage site could indicate a second site of initiation of translation, which may produce a cytosolic form of MnSOD starting with MGLT (highlighted in orange). (C) FeSOD lacks a potential targeting sequence. Metal‐binding sites are highlighted in red. NCBI accessions of each sequence are as follows: CuZnSODS (WP_015163683.1, BAC89922.1, WP_006457095.1, WP_011619688.1 and WP_015114498.1), MnSODs (AFY68900.1, QUX80117.1, WP_250121708.1, WP_073632316.1 and WP_010994247.1) and FeSODS (WP_015166022.1, WP_010997089.1, WP_010872652.1, WP_010469685.1 and WP_006041934.1).

MnSOD of *Pseudanabaena* sp. PCC7367 has a predicted signal peptide sequence containing a twin arginine motif (RR), followed by a hydrophobic segment and an AxA peptidase cleavage site (Figure 4A). This indicates that MnSOD may be a substrate of the TAT protein translocation system that transports proteins in their folded state into either the periplasmic space or the thylakoid lumen, or both. Homologues to the *E. coli* Tat system were also identified in *Pseudanabaena* sp. PCC7367, namely, two Tat A homologues and a single Tat C homologue, suggesting that it has a minimal Tat translocation system (Russo and Zedler 2021). The MnSOD protein sequence also carries a second methionine right after the predicted peptidase cleavage site, which may be an alternative start codon for a cytosolic form of MnSOD, starting with the sequence MGLT.

**FIGURE 5:**
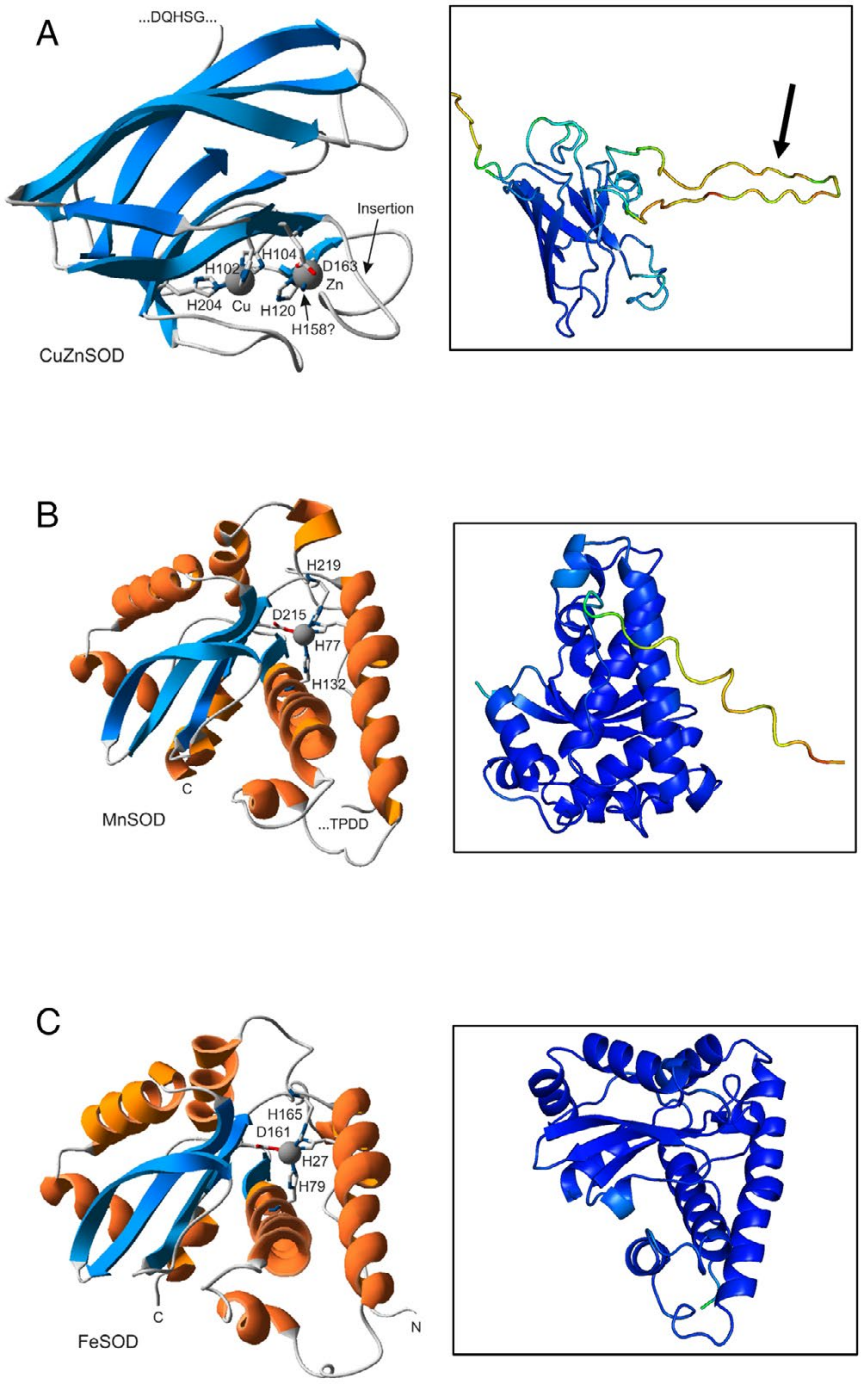
Predicted protein structures of superoxide dismutases encoded on the genome of *Pseudanabaena* sp. PCC7367. (A) The CuZnSOD protein prediction of the truncated protein cleaved at the AxA signal peptidase cleavage site. The arrows indicate 23 aa insertion. The N‐ternimal is cut off in this frame. (B) *Pseudanabaena* MnSOD cleaved at the signal peptidase cleavage site (AxA). (C) The structural predictions for the complete protein sequence for FeSOD. The models on the left were generated at the Phyre2 model server (Kelley et al. 2015) and visualised using the Swiss‐PDB viewer (Guex, Peitsch, and Schwede 2009). Figures on the right were generated using Alphafold2 (Jumper et al. 2021; Varadi et al. 2021) and shaded using PYMOL2 (DeLano 2020). Blue shading indicates high confidence in structure (100%) with decreasing confidence through the spectrum to deep red, indicating a confidence of 25%.

CuZnSOD has a positively charged N‐terminal sequence followed by a hydrophobic segment (Figure 4B), a classical signal peptide for the Sec translocation system. This suggests that CuZnSOD may be transported through the plasma membrane and/or the thylakoid membrane of *Pseudanabaena* sp. PCC7367 and hence be in the periplasmic space and/or the thylakoid lumen. The alignment of related protein sequences for cyanobacterial SodC highlighted a 23 amino acid insertion sequence, which may interfere with the binding of Zn^2+^ in the enzyme reaction center, thereby potentially reducing its activity (Figure 5).

FeSOD was found to have no positively charged N‐terminus and no hydrophobic signal, suggesting that it is a soluble cytoplasmic protein (Figure 4C).

The predicted protein structures using Alphafold 2 (Figure 5) indicated high confidence in the structures for FeSOD and the truncated MnSOD sequence generated to existing functional enzymes (Figure S6 for multiple sequence alignments), suggesting that they are indeed active in the cytoplasm and thylakoid/periplasmic space, respectively. The 23 aa insertion observed in the CuZnSOD sequence is not a common feature; however, the predicted structure of the truncated core enzyme has high congruence to existing CuZnSODs (Figure 5A). The insertion may, however, interfere with the binding of Zn in the active site, thereby possibly reducing its activity.

Furthermore, screening of the *Pseudanabaena* sp. PCC7367 genome indicated a complete complement of metal ion transporters to support the synthesis of MnSOD and CuZnSOD (Table S4). The iron transporters were previously identified in *Pseudanabaena* sp. PCC7367 (Enzingmüller‐Bleyl et al. 2022). Additionally, no genes encoding an SOR were found using tBLASTn, nor were they annotated as such in KEGG, so all superoxide inactivation must be a result of the SODs presented above. No homologue to the *E. coli* catalase gene was identified (Harada et al. 2021). However, the genome of *Pseudanabaena* sp. PCC7367 encodes five predicted peroxiredoxins, a presumably thioredoxin‐dependent glutathione peroxidase and one predicted heme‐containing peroxidase that could reduce the SOD dismutation product H_2_O_2_ (Table S5).

## 4 Discussion

Life on early Earth, prior to the oxygenation of the atmosphere during the GOE, was different to that found today (Fischer, Hemp, and Valentine 2016; Sessions et al. 2009). Cyanobacteria, the only known prokaryotes capable of conducting oxygenic photosynthesis, are considered primary agents for bringing about this change through the release of free O_2_ to the atmosphere by the photocatalytic hydrolysis of water (Cardona et al. 2019). The increase in levels of free oxygen, and its accompanying superoxide radical, raised the question as to whether early cyanobacteria were able to inactivate O_2_^•−^ arising from oxygenic photosynthesis (Fischer and Valentine 2019; Hamilton 2019). The phylogenetic trajectory of CuZnSODs matches that of the deep branching cyanobacteria at the base of their phylogenetic tree, suggesting that these early strains were able to process O_2_^•−^ prior to the GOE (Boden et al. 2021; Harada et al. 2021; Ślesak et al. 2019). Elevated levels of O_2_ per Chl a were recorded for a deep branching, CuZnSOD encoding cyanobacterium, *Pseudanabaena* sp. PCC7367, under an Archean simulated, anoxic atmosphere (Herrmann et al. 2021). Hence, in this study, we explored whether atmospheric oxygen levels influence the growth of cyanobacteria, and whether increased oxygen production is accompanied with increased levels of expression and activities of SODs, specifically CuZnSOD.

This study revealed a significantly increased growth rate for *Pseudanabaena* sp. PCC7367 cultured under an anoxic atmosphere, accompanied by significant increases in both viability markers of glycogen and protein. Additionally, there was no significant difference in accumulation of dissolved oxygen in the medium of stationary cultures during the light phase between all three atmospheres tested; however, significant differences were recorded during the dark phase between the Archean simulation and current LC conditions. Agitation of the cultures simulated natural, shallow‐water oxygen oases described in the literature (Crowe et al. 2013; Eickmann et al. 2018; Herrmann et al. 2021; Homann et al. 2015), suggesting that early cyanobacteria would not have been subjected to high O_2_ levels in the dark, allowing them time to recover from light‐induced photosynthetically generated free radicals.

Cyanobacterial Chl a content is commonly used to assess filamentous cyanobacterial growth (Li et al. 2014), while the carotenoid content provides an indication of potential light stress in cyanobacteria capable of synthesising this pigment (Herrmann and Gehringer 2019). Additionally, carotenoids dissipate excess light energy, thereby scavenging O_2_^−•^ radicals in the cytoplasm (Latifi, Ruiz, and Zhang 2009). As no significant change in the carotenoid content was observed, we can assume that carotenoid production was not upregulated to compensate for additional free radical removal.

Furthermore, this study demonstrates enhanced total expression of total *sod* (*soda*, *sodB* and *sodC*) in the culture material harvested towards day's end, corresponding to high levels of O_2_ in the medium under all three atmospheres investigated. This agrees with studies on *Synechocystis* under LC conditions (Kim and Suh 2005) and literature reporting O_2_^−•^ levels peaking near midday in surface waters in the ocean and ponds (Zinser 2018). Specifically, *sodB* and *sodC* expressions, encoding FeSOD and CuZnSOD, respectively, correlate significantly to the levels of dissolved O_2_ in the medium but not to cellular activity. In contrast, the expression of *sodA*, encoding MnSOD, correlates significantly to cellular SOD activity. The significantly increased SOD activity observed during the light phase in cultures growing under the Archean simulated anoxic atmosphere compared to modern‐day conditions corresponds to the increased O_2_ release rates per Chl a content recorded (Herrmann et al. 2021) in cultures of *Pseudanabaena* sp. PCC7367 grown under the same experimental conditions.

Transcription of *sodA*, *sodB* and *sodC* increased in agitated HC and LC cultures in 19 h compared to cultures under anoxic conditions, with no significant difference in cellular SOD activity observed. While transcription levels often do not correlate to translation rates or protein activity, due to mRNA stability or translational regulation, our data suggest that cyanobacteria are constantly challenged growing under modern‐day levels of oxygen. They consequently require inactivation of O_2_^−•^, even in the dark, when no oxygen is being released from photosynthesis. Impairment of photorespiration, the process whereby toxic byproducts of the oxygenase reaction of Rubisco are recycled to reduce fixed C loss from the cell, has been demonstrated to increase CO_2_ fixation in a freshwater, more recently diverged cyanobacterium, *Synechocystis* sp. PCC6803, under present‐day atmospheric conditions, supplemented with 50 mM NaHCO_3_ (Zhou et al. 2021). While daytime levels of dissolved O_2_ are similar for all our culture conditions, photorespiration may be reduced under anoxic conditions, when entering the dark phase, possibly contributing to the observed higher growth rates under the Archean simulated atmosphere (Hagemann and Kern et al. 2016). Further research is required to identify whether reduced photorespiration in an anoxic environment could enhance primary productivity or whether increased transcription of SOD encoding genes without the corresponding increase in cellular activity may suggest a higher enzyme turnover rate under oxic conditions.

As total *sod* (*sodA*, *sodB* and *sodC*) transcription levels did not correlate to total cellular SOD activity, we explored whether the encoded SOD isoforms could either be exported out of the cytoplasm to the thylakoid or periplasmic space or remain membrane‐embedded. The genome of *Pseudanabaena* sp. PCC7367 carries a single homologue of SecA, SecD, SecY, SecE and SecG, with two homologues of SecF (Table S3), suggesting that it carries a functional Sec export pathway (Russo and Zedler 2021). Homologues to the *E. coli* Tat system were also identified in *Pseudanabaena* sp. PCC7367, namely, two Tat A homologues and a single Tat C homologue, suggesting that it has a minimal Tat translocation system (Russo and Zedler 2021). The analysis of the three putative SOD isoforms encoded by *Pseudanabaena* sp. PCC7367 suggested that there were potentially two active SODs in the cytoplasm. The FeSOD protein, which carries no signal peptide, would remain in the cytosol. This is in agreement with the cytosolic location of FeSOD in *Synechocystis* (Ke et al. 2014; Kim and Suh 2005).

The synthesis of MnSOD could be initiated from a second translation initiation site to generate a protein that would remain in the cytoplasm. Additionally, the longer protein carries a twin arginine motif, similar to that found in *Chroococcidiopsis* (Napoli et al. 2021), indicating that it may be transported through the thylakoid and/or cell membrane to function in the thylakoid lumen or periplasmic space, respectively. Multiple forms of MnSOD have been isolated from the thylakoid membrane, lumen or cytoplasm (Herbert et al. 1992; Raghavan, Rajaram, and Apte 2013, 2015). Given that the expression of MnSOD did not correlate to external dissolved O_2_ levels in the media, we propose that the MnSOD is predominantly targeted to the thylakoid lumen to inactivate reactive O_2_^−•^ species at the site where they are generated during photosynthesis. A correlation between the cellular SOD activity and the expression of *sodA* might also point to a partial cytosolic localisation of MnSOD similar to *Nostoc* sp. PCC7120 (Raghavan, Rajaram, and Apte 2013, 2015).

The CuZnSOD from *Pseudanabaena* sp. PCC7367 has no secondary translation site and, as it carries a Sec translocation signal peptide, would therefore presumably always be transported across the cell and/or thylakoid membrane. In contrast, the *sodC*‐encoded protein from *Chroococcidiopsis* does not carry a signal peptide and is assumed to be cytosolic (Napoli et al. 2021). Given that the expression of *Pseudanabaena* sp. PCC7367 *sodC* strongly correlated to external medium O_2_ levels, we propose that this SOD isoform is exported across the cell membrane to reduce the levels of O_2_^−•^ in the periplasm.

Our investigation has highlighted that the deep‐branching *Pseudanabaena* sp. PCC7367 has the genetic potential to target O_2_^−•^, whether from increased water hydrolysis in the thylakoid lumen, photorespiration in the cytoplasm or external atmospheric O_2_ diffusion into the periplasmic space. We appreciate that some of the traits we measure in *Pseudanabaena* sp. PCC7367 may be unique to this species and not present in other cyanobacteria because it has been evolving largely independently of these species for billions of years. However, several of their traits reflect ancestral Archean features. For example, phylogenetic reconstructions estimate that crown cyanobacteria lacked scytonemin biosynthesis genes and had *sodC* similar to *Pseudanabaena* sp. PCC7367 (Tamre and Fournier 2022; Boden et al. 2021). Furthermore, *Pseudanabaena* species have small cell sizes similar to Archean cyanobacteria (Sánchez‐Baracaldo 2015) and their thylakoids are in a parietal arrangement, which has been inherited from the most recent common ancestor that they share with the Nostocales (Mareš et al. 2019), which was present several billion years ago (Sánchez‐Baracaldo 2015; Sánchez‐Baracaldo et al. 2017; Fournier et al. 2021; Boden et al. 2021). These features are quite different from the fast‐growing, more recently derived lineages such as heterocyst‐forming strains. So, although *Pseudanabaena* sp. PCC7367 may differ from its Archean ancestors, it is reasonable to assume that some of its traits would have been present in early cyanobacteria.

In conclusion, this study demonstrates that the early branching cyanobacterium, *Pseudanabaena* sp. PCC7367, exhibits increased SOD activity when medium O_2_ levels were at their highest, under the anoxic atmospheric conditions simulating early Earth, prior to the GOE. Additionally, cultures grown under anoxic conditions grow faster and contain significantly more protein and glycogen, thereby contributing valuable biologically available C and N to the environment compared to cultures grown under modern‐day oxic conditions. These data show that aquatic marine cyanobacteria growing under present‐day atmospheric levels of O_2_ are exposed to significantly higher levels of dissolved oxygen under darkness, thereby potentially placing a higher O_2_^−•^ load on the cellular metabolism. In summary, this investigation helps us understand the complex interplay between O_2_ production, O_2_^−•^ and cellular protection mechanisms in deep‐branching cyanobacteria, offering valuable insights into the evolutionary mechanisms that shaped our planet's biosphere. Further investigations into the generation of reactive oxygen species and their location inside the cyanobacterial cell, together with studies into the functionality of SOD isoforms, will help us understand how these ancient organisms navigated the transition to O_2_‐rich environments. Additionally, by further researching the evolution of photorespiration, we will gain significant insights into the foundations for the arrival of diverse plant life forms that thrive in today's oxygenated atmosphere.

## Conflicts of Interest

The authors declare no conflicts of interest.

## Supporting information


Data S1.

Data S2.

Data S3.

## Data Availability

All data generated and presented in this publication are available in Supporting Information.
