# Supplementary material for: Early‐Branching Cyanobacteria Grow Faster and Upregulate Superoxide Dismutase Activity Under a Simulated Early Earth Anoxic Atmosphere
Source: Geobiology. 2024 Dec 12;22(6):e70005. doi: 10.1111/gbi.70005 (PMC11636452; doi:10.1111/gbi.70005)
Supplement: Supplementary file 2 — Data S2. [file GBI-22-e70005-s003.pdf]

Supplementary Tables for the paper entitled:

Early-branching cyanobacteria grow faster and up-regulate superoxide dismutase activity under a simulated early Earth anoxic atmosphere.

Supplementary Table 1: Primer sequences. Primers specific for the superoxide dismutase genes, *sodA* (MnSOD), *sodB* (FeSOD) and *sodC* (Cu/ZnSOD), identified in *Pseudanabaena* sp. PCC7367 were designed as indicated below, as well as the reference gene primer pair targeting the *rpoC1* gene encoding the RNA polymerase beta subunit (Enzingmüller-Bleyl et al., 2022).

Gene of interest	Region in genome CP003592.1	Corresponding protein (NCBI Accession)	Locus	Primer direction	Sequence (5´ → 3´)	Tm [°C]	GC content (%)	Product length in base pairs (bp)
sodA	770,159 to 770,920	WP_015163874.1	Pse7367_0596	Forward	GCGCTCATGCCTGCTAAATC	60.04	55	167
				Reverse	CCTGATGACCCGTTTACGCT	60.11	55	
sodB	3,547,029 to 3,547,598	WP_015166022.1	Pse7367_2813	Forward	ACTGGCGCTTTGGCTAGTAA	57.3	50	165
				Reverse	GGGATTTTCAGCGTTGCCAG	60.11	56.4	
sodC	505,866 to 506,558	WP_015163683.1	Pse7367_0398	Forward	GCTGCTATGGGAGTTGTGGT	60.04	55	198
				Reverse	TTGGAGGTGATCGTTGAGGC	60.04	55	
				809R	GCTTCGGCACGGCTCGGGTCGATA	69,5	66,7	
rpoC1	n/a	n/a	Pse7367_0455	Forward	TGTTGGGTAAACGGGTTGAC	57.3	50	136
				Reverse	CGAATCAGGCGATTAATCACAAA	57.31	39.1	

Supplementary Table 2: Primer binding efficiencies. The binding efficiency of the primers designed to detect the SOD genes were determined for both genomic DNA (gDNA) and copy DNA (cDNA) reverse transcribed from RNA extracted from *Pseudanabaena* sp. PCC7367.

Genes targeted for analysis are the *sodA* (MnSOD), *sodB* (FeSOD) and *sodC* (Cu/ZnSOD) genes with the *rpoC1* gene as reference gene (Enzingmüller-Bleyl et al., 2022). The primer efficiencies were determined using genomic DNA of *Pseudanabaena* sp. PCC 7367 ranging from 10 000 000 copies to 1 copy of the targeted gene, as well as dilutions of cDNA. All genes targeted were present in a single copy on the genome of *Pseudanabaena* sp. PCC 7367.

Gene of Interest	gDNA Dilutions	cDNA Dilutions	Primer Efficiency % with gDNA	Primer Efficiency % with cDNA
rpoC1	10 ng	10 ng	107.70	102.68
sodA	10 ng	10 ng	95.79	88.7
sodB	10 ng	10 ng	89.30	88.7
sodC	10 ng	10 ng	98.02	97.88

Supplementary Table 3: Secretory pathways encoded in *Pseudanabaena* sp. PCC7367. The presence of genes encoding putative ***sec*** (Russo & Zedler, 2021; Supplementary Tables S2 & S3) and ***tat*** (Russo & Zedler, 2021) (Supplementary Table S4) pathways were obtained through a similarity search using the characterised protein sequences obtained from *E. coli* K12 MI1665 or *Synechocystis* sp. PCC6803.

Gene name	**Bait organism**	**Bait gene**	***Pseudanabaena* PCC 7367 uniprotkb**	**BLASTp E value**
secA	*E. coli* K12 MI1665	P10408|SecA	K9SFH1	6.81e-63
secD	*E. coli* K12 MI1665	P0AG90|SecD	K9SFP1	1.34e-61
secF	*E. coli* K12 MI1665	P0AG93|SecF	K9SFP1	1.81e-06
secF	*E. coli* K12 MI1665	P0AG93|SecF	K9SEI8	2.52e-31
secY	*E. coli* K12 MI1665	P0AGA2|SecY	K9SHY3	1.44e-107
secE	*Synechocystis sp. PCC6803*	P38382|SecE	K9SDF3	1.46e-16
secG	*Synechocystis sp. PCC6803*	P74508|SecG	K9SFY9	5.96e-21
tatA	*E. coli* K12 MI1665	P69428|TatA	K9SKB5	2.24e-05
tatA	*E. coli* K12 MI1665	P69428|TatA	K9SI06	5.16e-05
tatC	*E. coli* K12 MI1665	P69423|TatC	K9SCK0	2.55e-27

Supplementary Table 4: Metal transporters in *Pseudanabaena* sp. PCC7367. The presence of metal transporters encoded by *Synechocystis* PCC6803 for the metal cofactors required for the SOD isoforms investigated in this study, is indicated in the table below (modified from Sharon *et al.*, 2014). The characterized protein sequences from *Synechocystis* sp. PCC6803 (NC_000911.1) were used to search for similarity homologues in the genome of *Pseudanabaena* sp. PCC7367 (NC_019701.1) using tblastn (Altschul, 1991).

**Metal**	***Gene name***	**Description**	***Synechocystis* 6803 NC_000911.1 Gene locus tag**	***Synechocystis* 6803 Protein ID**	***Pseudanabaena* PCC 7367 NC_019701.1 Gene locus tag**	**Description**	**BLASTp E value**
**Mn**	*mntC*	PsaA, metal ABC transporter substrate binding domain	SGL_RS08820	WP_010872546.1	WP_015163932.1	zinc ABC transporter substrate-binding protein	4e-44
	mntA	Inorganic Mn^{2+}/Zn^{2+} + ion transporter ATPase component	SGL_RS08815	WP_010872545.1	WP_015163931.1	metal ABC transporter ATP-binding protein	8e-59
	mntB	ABC 3 transporter permease	SGL_RS0881	WP_010872544.1	WP_015166116.1	ABC-type Mn^{2+}/Zn^{2+} transport system, permease	1e-59
**Zn**	*znuB*	ABC-type Mn^{2+}/Zn^{2+} transport system, ATP-binding protein	SGL_RS04625	WP_010871740.1	WP_015163931.1	ABC-type Mn^{2+}/Zn^{2+} transport system, ATPase	1e-43
	znuC	hypothetical	SGL_RS04630	WP_010871742.1	/		No hit
	znuB (ZntC?)	zinc ABC transporter substrate-binding protein	SGL_RS04620	WP_010871739.1	WP_015166551.1	zinc ABC transporter substrate-binding protein	3e-28
**Cu**	*ctaA*	putative Cu binding site & ATP binding site	SGL_RS12475	WP_010873238.1	WP_051038173.1	copper-translocating P-type ATPase	0.0
	pacS	copper-translocating P-type ATPase	SGL_RS05435	WP_010871897.1	WP_015164254.1	copper-translocating P-type ATPase	0.0

Supplementary Table 5: Potential genes involved in the reduction of H_2O_2, the product of SOD dismutation of $O_2^{-\bullet}$.

The presence of genes potentially encoding peroxidases or peroxiredoxins were obtained from the KEGG database (Kanehisa et al., 2022) for the genome of *Pseudanabaena* sp. PCC7367 (NC_019701.1). The presence of a glutathione synthase and glutathione reductase were also confirmed.

**Description**	***Pseudanabaena* sp. PCC7367 KEGG entry**	**Description**	**BLAST against the protein data bank and manual sequence inspection**
Peroxiredoxin	Pse7367_1682	thioredoxin-dependent peroxiredoxin	BCP-type enzyme (alternative reductants)
Peroxiredoxin	Pse7367_2013	thioredoxin-dependent peroxiredoxin	Prx1-type enzyme (thioredoxin as a reductant)
Peroxiredoxin	Pse7367_2293	thioredoxin-dependent peroxiredoxin	BCP-type enzyme (alternative reductants)
Redoxin domain protein	Pse7367_2374	thioredoxin-dependent peroxiredoxin	Prx5-type enzyme (alternative reductants)
1-Cys peroxiredoxin	Pse7367_3020	thioredoxin-dependent peroxiredoxin	Prx6-type enzyme (reductant unknown)
Oxidoreductases	Pse7367_2328	peroxidase	Heme-containing peroxidase
Glutathione peroxidase	Pse7367_3393	glutathione peroxidase	Trx-dependent GPx (thioredoxin as a reductant)
Glutathione synthase	Pse7367_3280		
Glutathione reductase	Pse7367_0547	NADPH-glutathione reductase	

Supplementary Table 6: Results of One-way repeated measure ANOVA and/or ANOVA on ranks with environmental treatment condition as factor and Post-Hoc all pairwise multiple comparison procedures using Turkey Test and/or Holm-Sidak method for biomass parameters (Fig. 2 Suppl Fig 1). Bold numbers indicate significant differences with minimum p≤0.05. Number of replicates = 3.

**One-Way repeated measure biomass parameter**						
	**One-way repeated measure ANOVA**			**ANOVA on ranks**		
	**F**	**p**	**DF**	**Chi-square**	**p**	**Df**
**Chlorophyll a**				**22,00**	**≤0.001**	**2**
**Carotenoid**	**8,443**	**0,002**	**2**			
**Chl a: carotenoid ratio**	1.642	0.219	2			
**Protein**			**2**	**15.048**	**≤0.001**	**2**
**Glycogen**	**7,01**	**0,004**	**2**			
**Growth**	**214,102**	**≤0.001**	**2**			

**Post-Hoc pairwise multiple comparison**						
	**LC vs. Archean**		**LC vs. HC**		**HC vs. Archean**	
	q/t	*p*	*q/t*	*p*	*q/t*	*p*
**Chlorophyll a**	**6.351**	**<0.001**	3.175	0.064	3.175	0.064
**Carotenoide**	**5.804**	**0.001**	3.15	0.087	2.655	0.167
**Protein**	**4.715**	**0.002**	1.109	0.713	**3.606**	**0.029**
**Glycogen**	**5.103**	**0.004**	1.328	0.622	**3.776**	**0.035**
**Growth**	**18.930**	**<0.001**	2.227	0.068	**16.703**	**<0.001**

Supplementary Table 7: Results of One-way repeated measure ANOVA with environmental treatment condition as factor for growth media oxygen concentration (Suppl. Fig.2). In case of significant differences, Post-Hoc analysis using Holm-Sidak method was applied to identify differing groups: Bold numbers indicate significant differences with minimum p≤0.05. Number of replicates = 3.

**One-Way repeated measure for growth media oxygen concentration**			
**Time after onset of monitoring**	**One-way repeated measure ANOVA**		
	**F**	***p***	**DF**
**2h**	3.399	0.137	**2**
**14h**	2.826	0.172	**2**
**18h**	**17.941**	**0.01**	**2**
**22h**	**7.411**	**0.045**	**2**
**19h stirred**	**125.449**	**<0.001**	**2**

**Post-Hoc pairwise multiple comparison**						
**Time after onset of monitoring**	**LC vs. Archean**		**LC vs. HC**		**HC vs. Archean**	
	t	*p*	*t*	*p*	*t*	*p*
**2h**	n.s.					
**14h**	n.s.					
**18h**	**5.988**	**0.012**	**2.872**	**0.045**	3.117	0.07
**22h**	**5.401**	**0.04**	2.102	0.388	3.299	0.161
**19h stirred**	**15.575**	**<0.001**	**5.289**	**0.006**	**10.286**	**0.001**

Supplementary Table 8 : Results of repeated measure two way ANOVA using environmental treatment condition (Arch, HC, LC) as well as sampling time (Fig. 3, A-D) as factor for SOD gene expression, SOD protein activity and dissolved O_2 in the growth media. In case of significant differences, pairwise multiple comparison procedure using Duncan's Method for treatment (A) and time (B) as factor was applied to identify the respective groups of parameters that differ significantly. Bold numbers indicate significant differences with minimum p≤0.05. Number of replicates = 3.

	**Treatment**		**Time**		**Treatment x Time**	
	F-value	*p*	*F-value*	*p*	*F-value*	*p*
**qPCR *sodA* (MnSOD)**	5,156	0,078	**9,183**	**0,004**	**24,669**	**<0.001**
**qPCR *sodB* (FeSOD)**	**105,979**	**<0.001**	**61,534**	**<0.001**	**27,317**	**<0.001**
**qPCR *sodC* (CuZnSOD)**	**125,71**	**<0.001**	**21,583**	**<0.001**	**6,632**	**<0.001**
**qPRC *sodABC* Sum**	**102,919**	**<0.001**	**22,448**	**<0.001**	**4,189**	**0,007**
**SOD enzyme activity**	**85,075**	**<0.001**	**540,712**	**<0.001**	**158,534**	**<0.001**
**Dissolved O_2**	**11,069**	**0,023**	**112,171**	**<0.001**	**3,167**	**0,024**
**Post-Hoc pairwise multiple comparison**						
**A) Factor Treatment**						
	**LC vs. Archean**		**LC vs. HC**		**HC vs. Archean**	
	q	*p*	*q*	*p*	*q*	*p*
**qPCR *sodA* (MnSOD)**	**4,534**	**0,035**	2,486	0,154	2,048	0,221
**qPCR *sodB* (FeSOD)**	**16,783**	**<0.001**	1,938	0,243	**18,721**	**<0.001**
**qPCR *sodC* (CuZnSOD)**	**22,423**	**<0.001**	**11,361**	**0,002**	**11,063**	**0,002**
**qPRC *sodABC* Sum**	**18,704**	**<0.001**	2,543	0,147	**16,162**	**<0.001**
**SOD enzyme activity**	**17,118**	**<0.001**	2,603	0,14	**14,514**	**<0.001**
**Dissolved O_2**	**6,652**	**0,01**	3,484	0,07	3,167	0,089
**B) Factor Time**						
	**qPCR *sodA* (MnSOD)**		**qPCR *sodB* (FeSOD)**		**qPCR *sodC* (CuZnSOD)**	
	q	*p*	*q*	*p*	*q*	*p*
14.000 vs. 18.000	0.0145	0.992	12.15	**<0.001**	0.187	0.898
14.000 vs. 19.000	4.041	**0.025**	19.701	**<0.001**	11.234	**<0.001**
14.000 vs. 2.000	0.0272	0.985	17.263	**<0.001**	4.438	**0.019**
14.000 vs. 22.000	4.521	**0.015**	16.668	**<0.001**	1.943	0.224

18.000 vs. 19.000	4.056	**0.028**	7.552	**0.001**	11.047	**<0.001**
18.000 vs. 2.000	0.0416	0.979	5.113	**0.008**	4.251	**0.02**
18.000 vs. 22.000	4.507	**0.013**	4.519	**0.013**	1.756	0.25
2.000 vs. 19.000	4.014	**0.022**	2.439	0.123	6.796	**0.002**
22.000 vs. 19.000	8.563	**<0.001**	3.033	0.074	9.291	**<0.001**
22.000 vs. 2.000	4.549	**0.017**	0.594	0.685	2.495	0.116
	**qPRC *sodABC* Sum**		**SOD enzyme activity**		**Dissolved O_2**	
	q	*p*	*q*	*p*	*q*	*p*
14.000 vs. 18.000	0.0145	0.992	12.15	**<0.001**	0.187	0.898
14.000 vs. 19.000	4.041	**0.025**	19.701	**<0.001**	11.234	**<0.001**
14.000 vs. 2.000	0.0272	0.985	17.263	**<0.001**	4.438	**0.019**
14.000 vs. 22.000	4.521	**0.015**	16.668	**<0.001**	1.943	0.224
18.000 vs. 19.000	4.056	**0.028**	7.552	**0.001**	11.047	**<0.001**
18.000 vs. 2.000	0.0416	0.979	5.113	**0.008**	4.251	**0.02**
18.000 vs. 22.000	4.507	**0.013**	4.519	**0.013**	1.756	0.25
2.000 vs. 19.000	4.014	**0.022**	2.439	0.123	6.796	**0.002**
22.000 vs. 19.000	8.563	**<0.001**	3.033	0.074	9.291	**<0.001**
22.000 vs. 2.000	4.549	**0.017**	0.594	0.685	2.495	0.116

Supplementary Table 9: Pearson's correlation of parameters monitored during 24 h sampling (Fig. 3). Bold numbers indicate significant differences with minimum p≤0.05. Abrev. r= correlation coefficient, n= number of samples. Number of replicates = 3.

		**qPCR *sodA***	**qPCR *sodB***	**qPCR *sodC***	**qPCR *sodABC* sum**	**SOD enzyme activity**	**medium O_2**
**Time**	r	0.0796	-0.0238	-0.00657	0.0209	-0.0844	-0.0417
	p	0.603	0.877	0.966	0.892	0.582	0.785
	n	45	45	45	45	45	45
**qPCR *sodA***	r		-0.149	**0.521**	**0.47**	**0.349**	0.234
	p		0.327	**0.000242**	**0.00114**	**0.0186**	0.122
	n		45	45	45	45	45
**qPCR *sodB***	r			**0.382**	**0.797**	0.071	**0.651**
	p			**0.00958**	**5.71E-11**	0.643	**0.00000127**
	n			45	45	45	45
**qPCR *sodC***	r				**0.725**	0.0142	**0.675**
	p				**1.79E-08**	0.926	**0.000000376**
	n				45	45	45
**qPCR *sodABC* sum**	r					0.242	**0.751**
	p					0.11	**2.81E-09**
	n					45	45
**SOD enzyme activity**	r						0.202
	p						0.183
	n						45

References:

Altschul, S. F. (1991). Amino acid substitution matrices from an information theoretic perspective. *Journal of molecular biology*, *219*(3), 555-565.

Enzingmüller-Bleyl, T. C., Boden, J. S., Herrmann, A. J., Ebel, K. W., Sánchez-Baracaldo, P., Frankenberg-Dinkel, N., & Gehringer, M. M. (2022). On the trail of iron uptake in ancestral Cyanobacteria on early Earth. *Geobiology*.

Kanehisa, M., Furumichi, M., Sato, Y., Kawashima, M., & Ishiguro-Watanabe, M. (2022). KEGG for taxonomy-based analysis of pathways and genomes. *Nucleic acids research*, *51*(D1), D587-D592. https://doi.org/10.1093/nar/gkac963

Russo, D. A., & Zedler, J. A. (2021). Genomic insights into cyanobacterial protein translocation systems. *Biological chemistry*, *402*(1), 39-54.
